# A two-phase approach for the identification of refugees with priority need for mental health care in Lebanon: a validation study

**DOI:** 10.1186/s12888-016-1154-5

**Published:** 2017-01-18

**Authors:** Augusto E. Llosa, Mark Van Ommeren, Kavitha Kolappa, Zeina Ghantous, Renato Souza, Pierre Bastin, Andrej Slavuckij, Rebecca F. Grais

**Affiliations:** 1 Epicentre, Paris, France; 2 Department of Mental Health and Substance Abuse, World Health Organization, Geneva, Switzerland; 3 MGH/McLean Psychiatry Residency, Harvard University, Boston, MA USA; 4 Operational Center Geneva, Médecins Sans Frontières, Geneva, Switzerland

**Keywords:** Screening, Validation, Psychometrics, Sensitivity, Mental health, Psychiatric, Emergencies, Humanitarian, Refugees

## Abstract

**Background:**

Time and resource efficient mental disorder screening mechanisms are not available to identify the growing number of refugees and other forcibly displaced persons in priority need for mental health care. The aim of this study was to identify efficient screening instruments and mechanisms for the detection of moderate and severe mental disorders in a refugee setting.

**Methods:**

Lay interviewers applied a screening algorithm to detect individuals with severe distress or mental disorders in randomly selected households in a Palestinian refugee camp in Beirut, Lebanon. The method included household informant and individual level interviews using a Vignettes of Local Terms and Concepts for mental disorders (VOLTAC), individual and household informant portions of the field-test version of the WHO-UNHCR Assessment Schedule of Serious Symptoms in Humanitarian Settings (WASSS) and the WHO Self Reporting Questionnaire (SRQ-20). A subset of participants were then reappraised utilizing the Mini International Neuropsychiatric Interview (MINI), WHO Disability Assessment Schedule II, and the Global Assessment of Functioning. The study constitutes a secondary analysis of interview data from 283 randomly selected households (*n* = 748 adult residents) who participated in a mental health disorders prevalence study in 2010.

**Results:**

The 5-item household informant portion of WASSS was the most efficient instrument among those tested. It detected adults with severe mental disorders with 95% sensitivity and 71% specificity (Area Under Curve (AUC) = 0.85) and adults with moderate or severe mental disorder with 85.1% sensitivity and 74.8% specificity (AUC = 0.82). The complete screening algorithm demonstrated 100% sensitivity and 58% specificity.

**Conclusions:**

Our results suggest that a two phase, screen-confirm approach is likely a useful strategy to detect incapacitating mental disorders in humanitarian contexts where mental health specialists are scarce, and that in the context of a multi-step screen confirm mechanism, the household informant portion of field-test version of the WASSS may be an efficient screening tool to identify adults in greatest need for mental health care in humanitarian settings.

**Electronic supplementary material:**

The online version of this article (doi:10.1186/s12888-016-1154-5) contains supplementary material, which is available to authorized users.

## Background

Both during and after humanitarian crises, the mental and psychosocial wellbeing of affected populations is at risk [1–4]. Exposure to potentially traumatic events is a potent risk factor for mental disorder [1–4]. Moreover, continued daily stressors, as well as disrupted access to social support mechanisms and mental health care-including for people with pre-existing mental disorders- may exacerbate the risk [1–4]. Even in stable and well-resourced settings, mental disorders incur large disability and societal costs and remain largely undertreated, notwithstanding the mounting evidence of the efficacy of interventions [5–8]. This well-known treatment gap is further complicated in crises by large numbers of people displaced by conflict, persecution, and generalized violence currently estimated at 59.5 million globally. Of these, 86% are hosted by low and middle income countries and at least 6.4 million are considered as long-term displaced such as those in protracted refugee settings [9].

Established in a Beirut suburb in 1948 to accommodate refugees fleeing the conflict in Galile, northern Palestine, the Burj el-Barajneh refugee camp hosts approximately 20,000 refugees, many of whom have only ever known life in the camp. The mostly Palestinian camp is besieged by social and economic exclusion [10, 11] and a host of daily life stressors including institutional and legal discrimination, placing residents at increased risk for mental distress and disorders [4, 12, 13]. For Palestinian in refugee camps in Lebanon, joblessness is estimated at 56% in general and 63% for those 15–65 years of age. More specifically in Burj el-Barajneh, only 10% of those employed hold professional positions or are senior officials or managers. The majority work in craft or related trade work [10]. Previously we reported on a 2010 population-based survey conducted in the Burj el-Barajneh refugee camp in Lebanon to assess the prevalence of mental disorders amongst camp residents, as well as to inform organizations providing mental health support in the camp and its surroundings. We estimated that one fifth of adults in the camp had at least one current mental disorder, and we found that these disorders were disabling and nearly ubiquitously untreated [14].

Detection of persons with priority needs for mental care in contexts like Burj-el-Barajneh remains challenging. Appropriate rapid and effective screening tools and strategies for people with mental disorders are often not available in crises. Self-report screening instruments are less reliable for low prevalence disorders such as psychosis,[15] while standard lay interviewer administered instruments may confound distress with mental disorders in settings where high levels of distress are to be expected [16, 17]. Due to contextual differences in the conceptualization and expression of distress and mental disorders, results from non-validated psychometric scales can be misleading [17, 18]. Nonetheless, given the scarcity of mental health professionals in humanitarian contexts, as well as the low recognition of moderate and severe mental disorders in general health care,[19] strategies and instruments for systematic detection are needed to ensure appropriate mental health care.

A multi-step screen-confirm approach which includes locally developed and also cross-culturally validated standardized tools may thus offer several advantages [16, 20, 21]. Stratified, two phase or double sampling screening strategies, such as the one presented here, likely increase efficiency, especially for low prevalence outcomes or when lengthy clinical assessments are not realistic [22, 23]. By utilizing locally derived, contextualized, and longer standardized questionnaires in the screening phase, we aimed to detect those in greatest need of mental health while avoiding the shortcomings of any one instrument type. By performing household and individual level, lay administered interviews in the first phase, we sought to reduce the screening burden, and to improve detection by taking into account multiple perspectives [21, 24]. Additionally, the two phase approach optimizes the use of a clinical psychologist by limiting their involvement to reappraisal of mostly screen positive subjects.

Here, we present a secondary data analysis describing the attributes of a screen-confirm strategy and its component instruments for the detection of mental disorders and related impairment, including those of a locally-derived instrument to detect severe disorders and the field test version of a new World Health Organization-United Nations High Commissioner for Refugees (WHO-UNHCR) tool for the detection of individuals with severe distress and impaired functioning.

## Methods

**Fig. 1:**
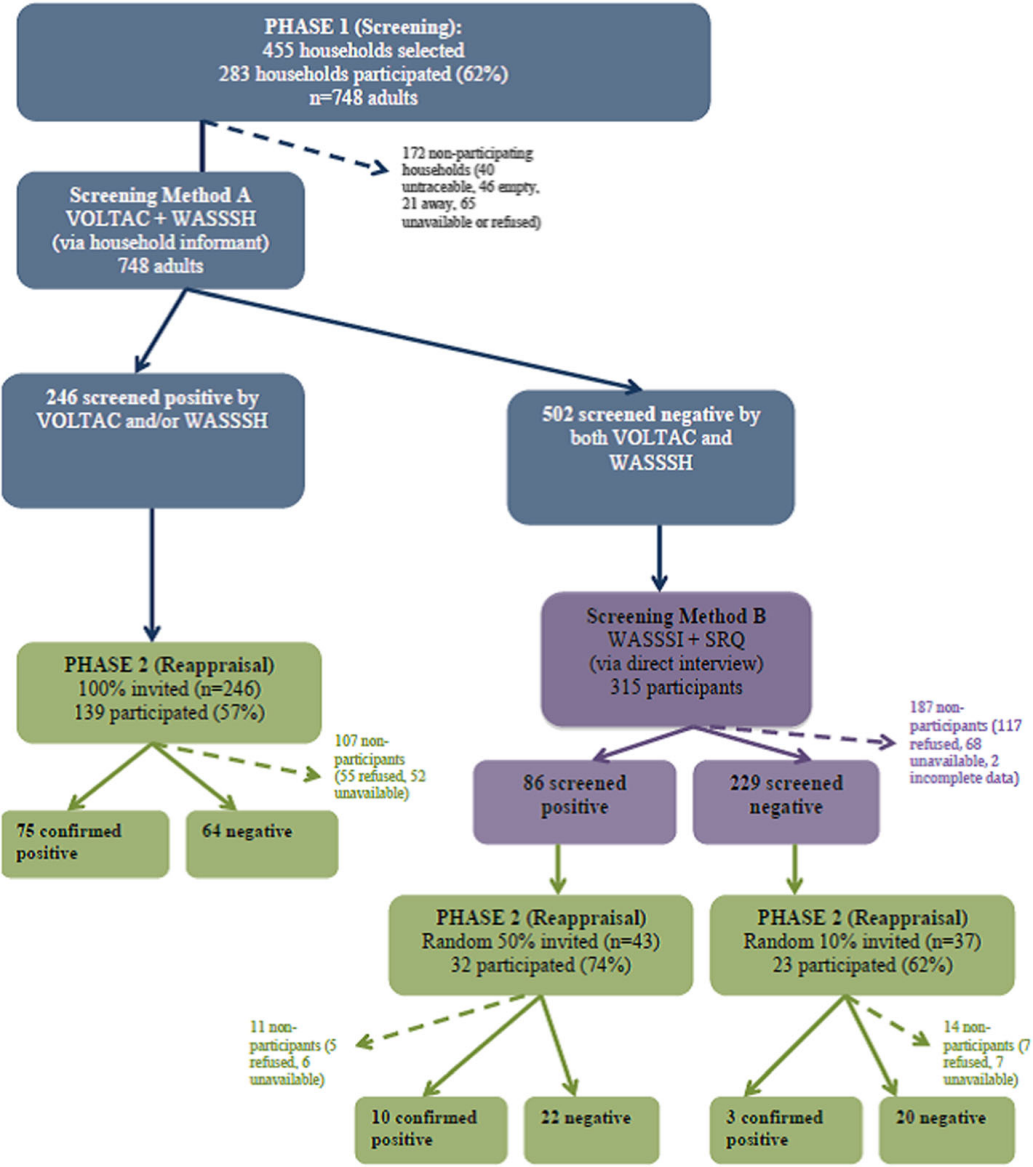
Subject flow diagram for parent study. This figure shows the flow of participants at each stage of the study

Information on the methods of our survey has been published previously [14]. Briefly, the survey adopted a two-phase (screen-confirm) strategy,[22, 23] in which the screening phase (phase 1) utilized four screening tools and two methods, namely, household informant and individual interviews. The confirmation phase (phase 2) utilized a clinical reappraisal of a subset of positive and negative cases to confirm diagnoses and assess degree of impairment (Fig. 1). We explore the psychometric properties of the four screening tools and of the overall strategy compared to a validated clinical interview and functioning measures.

### Participants

The survey sample consisted of all 748 adult residents from 283 households selected through simple random sampling from a comprehensive resident list provided by the refugee camp authorities. Clinical reappraisal was carried out on a subsample of 194 (Fig. 1).

### Instruments

#### Phase 1 Screening instruments

Four instruments were utilized during the screening phase, with two designated for each method. Screening Method A relied on household informants and utilized Vignettes of Local Terms and Concepts (VOLTAC) and the field-test version of the WHO-UNHCR Assessment Schedule of Serious Symptoms in Humanitarian Settings-Household Interview (WASSS-H) [25]. Screening Method B relied on individual interviews and utilized the field-test version of the WHO-UNHCR Assessment Schedule of Serious Symptoms in Humanitarian Settings-Individual Interview (WASSS-I) [25] and Self Reporting Questionnaire (SRQ) [26]. See Table 1 for additional information on these instruments.

**Table 1:** Instruments used to screen for mental disorders in phase 1

Instrument	Description
VOLTAC	With the aim of considering culturally relevant terms and expressions of mental disorders in the screening process, vignettes including local terms and concepts for mental illness were developed through focus groups with the camp residents. These concepts included examples of visual and auditory hallucinations, paranoia, self-talking, unsociability, poor appearance, and perceived abnormal behavior. VOLTAC was applied by reading vignette, broken down into its component sections and marking if the household representative identified any individual in their home with each particular item. Vignette components were: 1. Hears voices/sees things others don’t; 2. Complains someone is watching/following; 3. Seen talking to self often; 4. Not sociable/does not like to be with people; 5. Does not take care of appearance/hygiene; 6. Shows abnormal behavior.
WASSS	The WHO-UNHCR Assessment Schedule of Serious Symptoms in Humanitarian Settings [25] is a 2-part instrument designed to detect people with symptoms of severe distress and impaired functioning, and thus in priority need of mental health care. It is a brief, lay-administered instrument well adapted to humanitarian contexts; it is designed to sidestep the frequently confounded transient distress and mental disorders found in these settings, often posing an interpretative challenge when using diagnostic instruments.
- WASSS-H	The household informant portion of WASSS asks the respondent about the experiences of other household members (including symptoms of psychosis, convulsions, and functional impairment). It contains 7 questions eliciting dichotomous responses plus two open-ended questions. As our study was limited to adults at least 18 years or older two questions about children and adolescents were not asked, resulting in 5 items. Scoring positive on any item was considered to indicate a likely need for mental health care. This portion asks the household representative if anyone in their household was so distressed, disturbed or upset that he or she was: 1. completely inactive; 2. unable to carry out normal activities; 3. acting in strange ways; 4. not taking care of him/her self; 5. not caring for young children he/she is responsible for.
- WASSS-I	Individual interview portion of WASSS asks the respondent about severe, common distress symptoms and impaired functioning they have experienced themselves. It is comprised of 6 questions with 5 Likert-scale response options, indicating the frequency with which varieties of symptoms are felt. In our analysis, responses were reclassified with *all* and *most of the time* indicating a positive response, and *none of the time*, a *little of the time*, and *some of the time* indicating a negative response. The items are: 1. so afraid that nothing could calm you down; 2. so angry that you felt out of control; 3. so uninterested in things that you used to like that you did not want to do anything at all; 4. so hopeless that you wanted to be dead; 5. so severely upset about an event in your life that you tried to avoid places people, conversations or activities that reminded you of the event?; 6. unable to carry out essential activities for daily living because of these feelings
SRQ	The 20-item Self Reporting Questionnaire (SRQ) [26] is a WHO mental health screening instrument to detect mental disorders across different countries and cultures. It has been validated in many settings, including an Arabic version in Saudi Arabia and the United Arab Emirates [36, 37].

*VOLTAC* vignette of local terms and concepts, *WASSS*-*H* WHO-UNHCR assessment schedule of serious symptoms in humanitarian settings-household interview (WASSS-H), *WASSS*-*I* WHO-UNHCR assessment schedule of serious symptoms in humanitarian settings-household interview - individual interview portion, *SRQ*-20 self reporting questionnaire - 20 question version

#### Phase 2 Confirmatory instruments

Clinical reappraisal was carried out using the Mini International Neuropsychiatric Interview (MINI) [27] for making diagnoses, and two functional assessment scales to discern degrees of impairment (i.e. the Global Assessment Functioning (GAF) [28] and 12-question, interviewer-administered version of the WHO Disability Assessment Schedule-II (DAS-II) [29]).

Following confirmation of linguistic applicability in Lebanon, the Arabic version of the MINI,[30] was applied by a local, licensed psychologist to conduct clinical reappraisal in this study. The English version has been validated against the longer Structured Clinical Interview for DSM diagnoses (SCID) and Composite International Diagnostic Interview (CIDI), [27, 31] while the Arabic version has been validated against expert diagnosis [30]. Included in the assessment were: major depressive episode, dysthymia, suicidality, manic and hypomanic episode, generalized anxiety disorder, panic disorder, agoraphobia, social phobia, obsessive-compulsive disorder, posttraumatic stress disorder, psychotic disorders, and mood disorders with psychotic features. Some modules were excluded: alcohol and drug dependence, due low probability of obtaining forthright answers; eating disorders, due to the expected low prevalence in this setting; antisocial personality disorders due low probability of correct classification in single sitting. Diagnoses and related time periods (i.e., current, past, or recurrent episodes) were assigned according to the instrument’s instructions.

A local psychologist assessed symptom and disability severity in this study by utilizing two validated and widely used instruments, the Global Assessment of Functioning (GAF) [28] and the 12-question interviewer administered version of the WHO Disability Assessment Schedule-II (DAS-II) [28, 32]. Impairment was considered moderate for GAF scores between 51 and 60 or DAS scores equivalent to the sample 75th percentile (16.7–41.6), whereas impairment was considered severe for GAF scores lower than 51 or DAS scores above the 90th sample percentile (41.7) [33–35]. Severe mental disorder was defined as current psychosis or any current mental disorder accompanied by severe impairment. Moderate mental disorders included current mental disorder accompanied by at moderate impairment.

### Procedures

#### Phase 1 Procedures

For Method A, the household survey, a senior member of each participating household was interviewed with the locally developed VOLTAC vignette and the WASSS-H. Reading the VOLTAC concepts one at a time, household representatives were asked to name individuals in their home whom they felt matched concepts in the vignettes. They were then asked each of the questions of WASSS-H independently in relation to each household member. Those identified by a positive response to any VOLTAC or WASSS-H item were considered to have screened positive in the household survey. The rest, considered Method A screen negative were invited to individual re-screening in Method B. Those identified by a positive response to 6 SRQ items [36, 37] or 1 WASSS-I item were considered to have screened positive in the individual interview, Method B (Fig. 1).

#### Phase 2 Procedures

All cases screening positive by method A, a random 50% of cases screening positive by method B, and a random 15% of case screening negative by both methods were selected to be clinically reappraised. The subset participating in phase 2 was interviewed at their home or an alternative location proposed by the interviewee, by a local licensed clinical psychologist utilizing the MINI, GAF and DAS instruments.

All participants in both phases as well as non-participants were informed about the free services and invited to attend a mental health center supported by Médecins Sans Frontières.

### Statistical analysis

We present accuracy, sensitivity, specificity, positive predictive value, negative predictive values, Receiver Operating Characteristic (ROC) curves and corresponding Area Under the Curve (AUC) values, based on the subsample participating in phase 2 (*n* = 139 from Method A; *n* = 55 from Method B). These estimates are adjusted through probability weights for selection procedures (into Method B and Phase 2), participation rates by screening strata, and age/gender categories (i.e. the inverse of the sampling fraction for each category of gender, age group, and screening result: Method A positive, Method B positive, and overall screen negative) [22, 23]. Crude proportions, spearman correlation coefficients (ρ) and Cronbach alpha (α) for internal consistency are unweighted as calculations were performed on phase 1 data. Data were double entered using Epidata v.3.1 (Odense, Denmark). Statistical analysis was performed using STATA v.12.1 (College Station, Texas).

### Ethics, consents and permissions

The survey was approved by the ethics committees of Médecins Sans Frontières and the Lebanese American University. Written informed consent was required of all participants and included the permission to publish on results from anonymized, aggregate data.

## Results

**Fig. 2:**
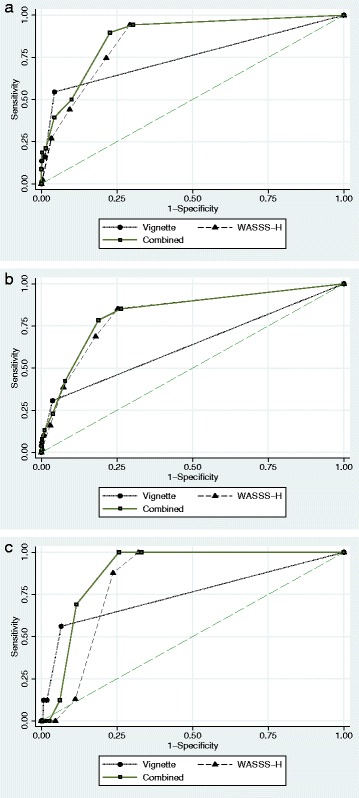
Receiver Operating Characteristic (ROC) Curves for Screening Instruments Utilized in Household Informant Interview Method for Different Outcomes. The ROC curve is a graphical plot illustrating the performace in terms of sensitivity and 1-specificity of the instrument at different cut off values, compared to the psycholgist’s assessment. **a** (*top*) shows the ROC for different cut-offs of the vignette (VOLTAC), the household informant component of WASSS (WASSS-H) and the combination of the instruments against the outcome of psychosis or of a current mental disorder accompanied by severe psychological impairment. **b** (*middle*) shows the ROC for the same instruments when predicting either psychosis or a current mental disorder accompanied by moderate or severe psychological impariment. **c** (*bottom*) shows the ROC for the same instruments when predicting only current psychosis

Given the nature of the items contained in the instruments, Method A results focus on severe disorders, while Method B results focus on moderate and severe disorders. Table 2 shows attributes of Method A and its instruments for the “severe” and “moderate or severe” mental disorders outcome. Table 3 shows attributes for Method B and its instruments for the outcome of “moderate or severe” mental disorders. Table 4 and Fig. 2, show method and instrument performance for a variety of outcomes.

**Table 2:** Household informant instrument performance for screening for mental disorders screening method-a (household informant)

	Responders		Severe Disorder^a^				Moderate or Severe Disorder^b^			
	Positive Response		Sensitivity	Specificity	PPV	NPV	Sensitivity	Specificity	PPV	NPV
VOLTAC Items	*n*	%	%	%	%	%	%	%	%	%
No positive responses	697	93.2								
At least 1 positive response	51	6.8	52.4	95.9	45.8	96.8	30.7	96.3	54.7	90.6
At least 2 positive responses	17	2.3	16.2	99.0	51.5	94.7	9.9	99.2	64.2	88.4
At least 3 positive responses	8	1.1	14.0	100.0	100.0	94.6	5.9	99.9	85.1	88.0
At least 4 positive responses	3	0.4	7.7	100.0	100.0	94.2	3.8	100.0	100.0	87.8
WASSS-H Items										
No positive responses	513	68.6								
At least 1 positive response	235	31.4	95.0	71.3	18.0	99.5	85.1	74.8	32.8	97.2
At least 2 positive responses	174	23.3	75.5	79.0	19.2	98.0	68.8	82.1	35.7	94.8
At least 3 positive responses	85	11.4	41.1	90.7	22.7	95.9	38.7	92.7	43.5	91.3
At least 4 positive responses	35	4.7	23.8	96.7	32.1	95.0	16.2	97.1	44.4	88.9
5 positive responses	4	0.5	2.2	99.6	28.3	93.9	2.1	99.8	55.0	87.5
Combined Method A Instruments										
No positive responses	502	67.1								
At least 1 positive response	246	32.9	95.0	70.3	17.3	99.5	85.1	73.7	31.9	97.1
At least 2 positive responses	190	25.4	90.9	77.9	21.4	99.2	78.4	81.2	37.6	96.3
At least 3 positive responses	94	12.6	50.7	90.3	25.8	96.5	42.3	92.2	43.9	91.7

^a^Severe disorders are current psychosis or any current mental disorder and severe impairment, *n* = 27

^b^Moderate to severe disorders are current severe disorders or any current mental disorder with at least moderate impairment, *n* = 53

Phase-1: *N* = 748; Phase-2: *N* = 194; proportions shown are probability weight adjusted for level of participation by screening stratum

*VOLTAC* vignette of local terms and concepts; *WASSS*-*H* WHO-UNHCR assessment schedule of serious symptoms in humanitarian settings-household interview (WASSS-H)

*PPV* positive predictive value, *NPV* negative predictive value

**Table 3:** Individual screening instrument performance for screening for mental disorders

Screening Method B (Individual Interview)	Phase 1 Participants		Moderate or Severe Disorder*			
Among Method A Screen Negatives	Positive Response		Sensitivity	Specificity	PPV	NPV
WASSS-individual questionnaire	*n*	(%)				
No most/all of the time responses	257.0	81.6	REFERENCE			
At least 1 most/all of the time responses	58.0	18.4	50.0	83.4	8.1	98.3
At least 2 most/all of the time responses	25.0	7.9	50.0	96.8	31.6	98.5
At least 3 most/all of the time responses	11.0	3.5	0.0	98.2	0.0	97.1
At least 4 most/all of the time responses	8.0	2.5	0.0	98.7	0.0	97.1
At least 5 most/all of the time responses	4.0	1.3	0.0	98.7	0.0	97.1
6 most/all of the time responses	1.0	0.3	−	−	−	−
SRQ-20						
No positive responses	112.0	35.6	REFERENCE			
At least 1 positive response	203.0	64.4	100.0	32.6	4.2	100.0
At least 4 positive responses	101.0	32.1	100.0	77.0	11.3	100.0
At least 6 positive responses	71.0	22.5	100.0	82.5	14.3	100.0
At least 7 positive responses	55.0	17.5	83.3	85.9	14.7	99.4
Combined Method B Instruments						
No positive responses	107.0	34.0	REFERENCE			
At least 2 positive responses	162.0	51.4	100.0	49.0	5.4	100.0
At least 6 positive responses	73.0	23.2	100.0	83.9	15.3	100.0
A least 1 WSSS or 6 SRQ positive responses	86.0	27.4	100.0	78.1	11.8	100.0

*Moderate-severe disorders are current severe disorders or any current mental disorder with at least moderate impairment, *n* = 53, Severe disorders are current psychosis or any current mental disorder and severe impairment, *n* = 27

Phase-1: *N* = 748; Phase-2: *N* = 194; proportions shown are probability weight adjusted for level of participation by screening stratum

*WASSS*-*I* WHO-UNHCR assessment schedule of serious symptoms in humanitarian settings-household interview - individual interview portion

*SRQ*-20 self reporting questionnaire - 20 Question version

*PPV* positive predictive value, *NPV* negative predictive value

**Table 4:** Screening method attributes for mental health outcomes

Combined Phase-1 Procedure^c^	Sensitivity	Specificity	PPV	NPV	Correctly classified	AUC
Disorder:	%	%	%	%	%	
Current severe disorder	100.0	53.6	12.5	100.0	56.5	0.77
Current moderate or severe disorder	100.0	57.5	25.4	100.0	62.9	0.79
Current mental disorder	100.0	62.4	39.0	100.0	69.7	0.81
Current moderate or severe impairment	83.2	53.3	14.1	97.2	55.8	0.68
Psychosis outcome						
VOLTAC^a^	56.1	93.4	8.3	99.5	93.0	0.75
WASSS-H^a^	**100.0**	**67.9**	3.2	100.0	68.3	0.84
Combined WASSS^a^	100.0	56.1	2.4	100.0	56.6	0.78
Combined method A^b^	**100.0**	**74.4**	4.0	100.0	74.7	0.87
Combined phase-1 procedures^c^	100.0	50.8	2.1	100.0	51.3	0.75

Considered screen-positive if positive by at least: ^a^1 response; ^b^2 responses, ^c^1 response from VOLTAC, WASSS-H, WASSS-I or 6 SRQ

*WASSS*-*I* WHO-UNHCR assessment schedule of serious symptoms in humanitarian settings-household interview-individual interview portion

*WASSS*-*H* WHO-UNHCR assessment schedule of serious symptoms in humanitarian settings-household interview-household informant portion

*SRQ*-20: self reporting questionnaire - 20 question version

*PPV* positive predictive value, *NPV* negative predictive value, *AUC* area under receiver operating characteristic curve

### Screening method A: VOLTAC and WASSS-H

The sample of 748 of participants from 283 households was 50.9% female with a mean age of 39 years (Standard Deviation, SD = 15.8) [14]. Overall, 246 of 748 adults (32.9%) screened positive by the household informant method, with 11 by vignette alone, 195 by WASSS-H alone, and 40 by both. Five hundred and two screened negative by both, of which, 315 (62.7%) participated in method B screening interviews and 86 (27%) screened positive. From Method A, 139 of 195 invited (56.5%) participated in phase 2, while from Method B 55 of 80 invited (69%) participated in the clinical reappraisal (Fig. 1).

### Results from VOLTAC

Of the 51 adults who screened positive by vignette, the average number of positive responses to the 5 items was 1.55 and ranged 1–4. Overall internal consistency of the vignette items was low (α = 0.60). The most common positive response was related to unsociability, followed by self-talking, then visual or auditory hallucinations. The most highly correlated items in the scale were unsociability with self-talking (ρ = 0.46), unsociability with paranoia (ρ = 0.39), and paranoia with hallucinations (ρ = 0.37). For the outcome of severe disorders, sensitivity was <22% for individual items. For the instrument, a cut off ≥1positive item had the highest screening performance for this outcome, with global sensitivity of 52.4%, specificity of 95.9% and AUC = 0.74 (Table 2).

### Results from WASSS-H

Of the 235 adults who screened positive by the household component of WASSS (WASSS-H), the average number of positive responses to the 5 questions was 2.3 and ranged between1-5. The overall internal consistency of the instrument was acceptable (α = 0.75). The most highly correlated items in the scale were distress-related inactivity with inability to carry out regular daily activities (ρ = 0.79) and with not caring for his/her self (ρ = 0.47); the latter two were also moderately correlated (ρ = 0.47), as were not taking care of self and not caring for children for whom he/she is responsible for (ρ = 0.43).

Distress-related inactivity was the most common response and showed the highest sensitivity (90.2%) and specificity (78.0%) against the outcome of severe mental disorder. A cut off of at least one positive response provided the highest sensitivity, specificity and AUC for this outcome: 95%, 71.3% and 0.85, respectively. Using the same cut off, for the moderate or severe mental disorder outcome, sensitivity was 85.1%, specificity was 74.8 and AUC was 0.82 (Table 2). While not the outcome of focus in this article, for any current mental disorder, sensitivity was 100%, specificity was 67.9% and AUC was 0.84. Sensitivity, specificity and AUC for specific disorders were much lower, likely due to small sample sizes, comorbidities and the nonspecific nature of the WASSS (data available on request).

### Results from combined method A instruments

The internal consistency of the combined household informant instruments was similar to that of WASSS-H alone (α = 0.72), with highest inter-instrument correlation between not caring properly for those they are responsible for (WASSS-H) and unsociability (ρ = 0.28) or self-talking (ρ = 0.21) (VOLTAC). Correlations between other questions were lower than 20%. Combined, such that a single positive response in either instrument was considered a screen-positive, Method-A performed similarly to WASSS-H alone, with 95.0% sensitivity, 70.3% specificity and AUC of 0.88 for the severe psychopathology outcome (Table 2).

### Screening method B: WASSS-I and SRQ

Method B of phase 1 offered a second screening opportunity for those who did not screen positive by Method A. Of 502 eligible adults, 315 (62.7%) participated and 86 (27.3%) screened positive by individual interviews; 17 screened positive by the individual portion of WASSS (WASSS-I) alone, 30 by SRQ-20, and 41 by both; 227 screened negative by both instruments. 55 of 80 (68.8%) selected to undergo phase 2 accepted and completed reappraisal procedures. The overall, adjusted prevalence of current mental disorders was 19.4% (12.6–26.2) [14].

Performance of Method B instruments could not be assessed for the outcome of severe disorders since 54 of 55 participants with this outcome screened positive by Method A, consequently skipping Method B assessments for direct confirmation in Phase 2. Performance of Method B is thus assessed for any remaining severe mental disorders and any moderate mental disorders.

### Results from WASSS-I

Internal consistency of WASSS-I was moderate (α = 0.78). The highest correlation was found for questions related to fear and anger (ρ = 0.49), followed by inability to carry out essential activities for daily living with questions related to disinterest (ρ = 0.44), hopelessness (ρ = 0.46) and avoidance (ρ = 0.43). Individual WASSS-I items showed sensitivity <33%, though combined and using a cut off ≥1 “most or all of the time” responses, the instrument performed better: 50.0% sensitivity, 83.4%specificity and AUC of 0.67 (Table 3).

### Results from SRQ

Internal consistency of the SRQ-20 instrument in our sample was good (α = 0.87). The a priori cut off of at least 6 positive responses yielded 100% sensitivity and 82.5% specificity, with an AUC of 0.91 to detect any remaining severe mental disorders or any moderate mental disorders (Table 3).

### Results from combined method B instruments

The combination of WASSS-I and SRQ did not demonstrate an advantage over the SRQ alone when predicting moderate to severe disorders in phase 2. Using the criteria of WASSS-I score ≥ 1 or SRQ-20 ≥ 6, corresponding sensitivity and specificity for remaining severe mental disorder or any moderate mental disorder were 100% and 78.1%, respectively, with AUC = 0.89 (Table 3).

The remaining cases of severe psychopathology participating in Method B interviews screened positive by SRQ but not WASSS-I. While not the outcome of focus in this article, Method B detected any current mental disorder with 100% sensitivity and 83.1% specificity, with AUC = 0.92.

### Combined screening instruments (methods A and B)

Combined phase 1 screening procedures predicted moderate or severe current mental disorder with 100% sensitivity and 57.5% specificity. Table 4 shows the screening method’s performance against several phase-2 confirmed diagnoses. For the psychosis outcome WASSS-H was 100% sensitive but specificity increased by 9.6% (from 67.9 to 74.4%) when combined with VOLTAC.

## Discussion

Our study suggests that a two phase, screen-confirm approach to detect moderate or severe mental disorders could be a useful strategy in humanitarian contexts where mental health care needs are large and mental health care is made available.

The complete phase 1 procedure detected 100% of subjects with moderate or severe mental disorders. The relatively lower specificity observed, 58%, is in part explained by the multifaceted screening approach which may have led to a relatively blunt procedure aimed to maximize sensitivity. A portion of the false positives for this outcome were individuals with common mental disorders which the parent prevalence study also aimed to detect.

While additional testing in other contexts is necessary, results also suggest that the household informant portion of the field-test version of the WASSS may be an efficient screener to identify those in greatest need for mental health support in humanitarian settings. Despite being designed to help identify individuals in priority need of mental health care in acute crises, the instrument performed well in this protracted refugee setting. In the context of a two part screening methodology, this 5-item household informant interview detected 95% of cases of active psychosis or other mental disorders accompanied by high levels of impairment, with 71% specificity, when applying a cut off of scoring positive on only one of the instruments’ 5 dichotomous items. Criterion validity was also demonstrated by discriminating severe psychopathology, including psychotic symptoms, and moderate or severe mental disorders (AUC = 0.85 and 0.82 respectively). Although test-retest reliability was not assessed, internal consistency of items (α = 0.75) suggests moderate reliability. Results are comparable to those shown by the widely used-but more difficult to score-screening scale for serious mental illness (K6) which showed AUC scores of 0.86 in Lebanon and ranged from 0.76 to 0.89 in 14 countries in the WHO World Mental Health Survey Initiative [38].

The individual portion of the field-test version of this instrument (WASSS-I) did not perform well against confirmatory assessment of moderate or severe mental disorder (AUC = 0.67). The SRQ performed much better, likely due to the broader underlying structure of this lengthier instrument. It is important to note that to reduce the testing burden on participants the original prevalence study, these instruments were applied only to those who did not screen positive during the household informant method. It is possible that results could differ if they were applied directly to all subjects.

Despite being locally derived, VOLTAC alone was less sensitive than anticipated, failing to identify nearly half of the people with the most severe conditions. On inspection, the vignette might have performed better if it included mood-oriented question assessing anger or hopelessness. As the instrument was created based on community focus groups, it suggests a possible mismatch between what the population and a clinical psychologist utilizing standardized instruments identify as evidence of severe mental illness and impairment. Using a similar vignette methodology to proactively identify people with mental disorders by community informants, a recent study in Nepal had more promising results for detecting people with mental disorder [39]. The VOLTAC likely requires further fine-tuning of culturally appropriate descriptions to improve recognition of mental disorder and may possibly do better if used by community health workers. Together, these findings stress the possibility of including culturally relevant terms and concepts in assessments, but also suggest that further methodological work is needed to understand why the VOLTAC did not perform well in contrast to the approach used in Nepal. These results highlight the advantages of applying standard measures in emergency and other contexts where there is often insufficient time to develop and validate culturally specific instruments. It is noteworthy, however, that combining the VOLTAC with the WASSS-H improved the performance of detection of psychosis (AUC = 84 to AUC = 87). This is likely explained by concepts in VOLTAC addressing symptoms most closely associated with psychosis such as hallucinations and delusions. Several key limitations are of note. Exclusion of some modules of the MINI (e.g. substance abuse and personality disorders [14].) could have affected instrument performance estimates, for instance driving down the positive predictive value if they were picked up on screening but not on confirmation due to omission of the corresponding modules. Non-participation and loss to follow-up may have introduced bias if those not completing the battery of tests differed in terms of symptomatology detectable by lay administered tests. Sensitivity could be falsely elevated, for instance, if those with conditions for which screening instruments and procedures were less well suited to detect were more likely to not participate in the second phase. To reduce testing burden on participants, those screening positive by the household informant method proceeded directly to clinical reappraisal [14]. Consequently, test-retest and inter-rater reliability were not ascertained, and instruments based on individual interviews could not be thoroughly, independently assessed. Individual instrument performance can only be interpreted in the context of this multistep approach.

Screening instruments demonstrated low positive predictive values in this context. For the more severe disorders, this is in part explained by their lower prevalence. The design also had an effect, however. As phase 1 procedures aimed to maximize detection of all those with current mental disorders and high levels of impairment through the use of multiple instruments and methods, the resulting high false positive ratio drove down specificity and positive predictive values. Using higher cut-offs should help improve these values.

While testing occurred in the context of a protracted refugee setting, many of the same daily life stressors are present as in acute crises such as conflict and disasters. The prevalence of transient distress and that of common mental disorders varies according to context, but the ability of these instruments to detect severe mental disorders should not be affected. Nonetheless, additional testing of key instruments in a greater variety of contexts would further contribute to our understanding of instrument performance and highlight areas for future research.

For the purposes of this study, only a fraction of screen positives and negatives needed to be clinically reappraised to determine the prevalence of mental disorders. If the aim is treatment in busy outpatient department and polyclinics such as those in humanitarian settings for instance, reappraisal of a random selection of subjects from different screening strata would not serve the purpose. Instead, screening instrument cut offs or thresholds would need to be adjusted according to program objectives and capacity and all screen positives reappraised. The benefit would be two-fold, in utilizing non-specialized personnel for screening purposes, while increasing the probability that people with severe mental disorders would receive needed mental health care. Screening should always be used in combination with service delivery [40].

## Conclusion

Our results suggest that a two phase, screen-confirm approach is likely a useful strategy to detect incapacitating mental disorders in humanitarian contexts where mental health specialists are scarce. Our results also suggest that in the context of a multi-step screen confirm mechanism, the household informant portion of field-test version of the WASSS may be an efficient screening tool to identify adults in greatest need for mental health care in humanitarian settings like Burj-el-Barajneh.

## Additional files

Additional file 1: “data leban”, containing study dataset as excel (.xls) file. (XLS 333 kb)
Additional file 2: “leban data dictionary”, containing variable names and code. (DOCX 15 kb)

## Abbreviations

AUC: Area under the curve
CIDI: Composite international diagnostic interview
DAS: Disability assessment schedule
GAF: Global assessment of functioning
MINI: Mini international neuropsychiatric interview
NPV: Negative predictive value
PPV: Positive predictive value
ROC: Receiver operating characteristic
SCID: Structured clinical interview for DSM diagnoses
SD: Standard deviation
SRQ: Self reporting questionnaire
UNHCR: United Nations high commissioner for refugees
VOLTAC: Vignettes of local terms and concepts
WASSS: Assessment schedule of serious symptoms in humanitarian settings
WASSS-H: Assessment schedule of serious symptoms in humanitarian settings - household interview
WASSS-I: Assessment schedule of serious symptoms in humanitarian settings - individual interview
WHO: World health organisation
α: Cronbach alpha
ρ: Spearman correlation coefficients ρ
