# Supplementary material for: A two-phase approach for the identification of refugees with priority need for mental health care in Lebanon: a validation study
Source: BMC Psychiatry. 2017 Jan 18;17:28. doi: 10.1186/s12888-016-1154-5 (PMC5241938; doi:10.1186/s12888-016-1154-5)
Supplement: Additional file 2: — “leban data dictionary”, containing variable names and code. (DOCX 15 kb) [file 12888_2016_1154_MOESM2_ESM.docx]

| **Participants** | |
| --- | --- |
|  |  |
| idm | Id number |
| one | Phase1 method A (HH informant method) |
| cmdpos | Phase1 method B (individual method) |
| p2 | Phase2 participants (confirmatory testing) |
| x2 | Random half of dataset |
| *fw* | *weight* |
|  | |
| ***Method A Instruments (household informant)*** | |
| **VOLTAC** | |
| smh01-smh06, smh09 | Items original scoring (yes/no/refused) |
| tsmh01-tsmh06, tsmh09 | Items binary scoring (yes/no) |
| smhpos | Overall score yes/no |
| tsc1-tsc4 | Cumulative score cutoffs for AUC |
|  |  |
| **WASSS Household** | |
| b41, b51, b61, b91, b101 | Items original scoring (yes/no/dk/refused) |
| tb1-tb5, tb12 | Items binary scoring (yes/no) |
| bpos | Overall score yes/no |
| tbc1-tbc5 | Cumulative score cutoffs for AUC |
|  |  |
| **Combined Method A** |  |
| sbpos | Cumulative score for AUC |
| mac0-mac8 | Cumulative score cutoffs for AUC |
| ***Method B Instruments (individual interview)*** | |
| **WASSS Individual** |  |
|  | Items original scoring (yes/no/dk/refused) |
|  | Items binary scoring (yes/no) |
|  | Overall score yes/no |
|  | Cumulative score for AUC |
|  |  |
|  |  |
|  | SRQ20 |
|  | Combined Medhod B instrumetns |
| washi | Combined WASSS |
|  | Outcomes |
| minipos | any mental disorder |
| cur | Any mental disorder (current) |
|  | moderate/severe impairment (current) |
|  | mental disorder (current) |
| decSFD | Severe Disorder (current) |
| decMFD | Mod+Sev Disorder (current) |
| curpsy / psy3 | Psychosis (current / lifetime) |
